# The Noise Nuisance Again

**Published:** 1895-10

**Authors:** 


**A Monthly Review of Medicine and Surgery.**

*EDITORS:*

THOMAS LOTHROP, M. D. - - WM. WARREN POTTER, M. D.

All communications, whether of a literary or business nature, should be addressed to the managing editor: 284 FRANKLIN STREET, BUFFALO, N. Y.

VOL. XXXV. OCTOBER, 1895. No. 3.

### THE NOISE NUISANCE AGAIN.

WE HAVE, on several occasions, called attention in these columns to the influence upon health of a class of noises that is as unnecessary as it is exasperating, as well as unhealthful. We return to the subject, because we feel it a duty to point out to the municipal authorities, from time to time, whatever is being permitted, either through neglect or indifference, to interfere with the health, comfort or happiness of our citizens. The rapid growth of Buffalo during the past ten years, with the prospect of a still greater increase during the next decade, makes it all the more necessary to reduce the noises connected with traffic to a minimum and to suppress all such as are unnecessary.

The fog-horn, that for two years played havoc with health and comfort, has been checked in its riotous and unseemly course, until now it falls within the range of toleration. We believe this modified screeching of the fog-whistle has been brought about through the timely offices of Mayor Jewett. We trust that he will address himself with equally satisfactory results to the steam-whistle nuisance, which is getting beyond the range of toleration.

The mayor's kind offices may, with propriety, begin by suppressing the whistle of the police boat, which comes nearer, if tradition is to be relied upon, to the noise of an infernal machine than anything yet invented. This whistle is so absolutely and totally unnecessary, that it should be stopped at once, and in this case the mayor possesses the power to act and to succeed.

The screeching of factory whistles and the tooting of locomotives is a matter that may properly fall within the province of the mayor to control, and we have no doubt it could be done by means of a circular letter addressed to the offenders. Factory whistles should be suppressed and the locomotive whistles restrained within the mere necessity for proper signals. The *Buffalo Express*, in its issue of September 9, 1895, has befittingly spoken on this subject, from which we quote as follows :

STEAM-WHISTLE NUISANCE.—THERE IS AN ORDINANCE PROHIBITING THE TOOTING OF FACTORY WHISTLES, BUT IT IS A DEAD LETTER.

In some sections of the city the annoyance from the blowing of whistles in factories at times becomes unbearable. The people living in a section built up by factories at times find the blowing of whistles a benefit, as it tells the housewife of the time to have meals ready, and in most cases is relied on to call her attention to the time that the head of the house is to be awakened to go to his daily toil. Sometimes, however, the people in charge of factories, by the use of a screeching whistle or the long, continuous blasts, make the whistle an unbearable nuisance.

A few years ago, when Justice Daniels was on the bench of the Supreme Court, the noon whistles of the factories on the Terrace were looked upon as an unmitigated nuisance and when Sheriff Lamy, at that time a deputy sheriff, was called before the court and was ordered to notify the manufacturers to stop whistling, he had an idea that such action would be in a measure beyond his authority. He was an officer of the court, however, and obeyed the order of the justice, placing the matter in a pleasant way, telling the factory owners of the annoyance the whistles were to the court. The owners of the establishments recognised the annoyance and willingly complied with the request. There was no further complaint from that source. In other sections of the city, however, there has been no stoppage, and in many cases the amount of blowing is almost unbearable. It might be well for the owners of factories to remember that there is an ordinance bearing on the subject.

Section 19 of chapter 9 of the ordinances reads : "No person, firm or corporation, owning or controlling any engine or boiler attached to which is a steam whistle, shall permit such whistle to be blown within the limits of the city, except upon vessels, crafts or floats in Buffalo harbor and river and the waters connected therewith ; nor shall any such vessel, craft or float use such whistle except for the purpose of giving the necessary marine signals. Any person, firm or corporation violating the provisions of this section, shall be subject to a penalty of $10 for each and every offense."

The ordinance has been in force for many years, but a conviction under it cannot be recalled.

Another noise nuisance that has lately grown up in Buffalo, is one for which the street commissioner is directly responsible. We refer to the present methods of collecting garbage. These men appear at an early hour in the morning with a rattling truck, that makes an ugly reverberating sound on the flagging in front of and between the dwellings, which is well-nigh intolerable. Directly after the garbage barrels are wheeled into the streets by means of this noisy truck, along come the teams, when the teamsters begin to shout at the horses and so, between the chattering of the men, the rattling of the trucks and the noisy teamsters, there is no peace from 6.30 to 8.00 A. M.

In the residence section of a city the garbage collectors should not begin their work before eight o'clock in the morning. They should conduct their work in as quiet a manner as possible, and if trucks are allowed they should have wood or hard rubber wheels. The street commissioner should be a man of discipline himself, capable of enforcing it in his subordinates.

The *Medical Record*, in its issue of July 19, 1895, devoted its leading editorial to the consideration of unnecessary noises, as follows :

The clamor of traffic is in some respects a necessity of the struggle for living and gain. The useless accessories of such a situation are too numerous to mention. Take, for instance, the numerous street cries of peddlers, the roar of the elevated train, the clang of the cable car, the irritating factory whistle, the intrusive hand-organ and even the reverentially suggestive church-bell.

Not long since, a piano fiend in an adjoining house rendered miserable the dying hours of an honored judge in brazen defiance of frequent protests from his bereaved family. We wonder if it be true, as the Swedenborgians assert, that pianos will be allowed in heaven. If so, it may be possible to assume that they will be tuned to a harmony unknown and unrecognisable by earthly mortals. In any event the only hope will be that the practising miss with her dreary and exasperating finger drill may not die before she is well started with a tune. The piano, however, has done more to educate humanity into a forced toleration of disagreeable noises than any other known agent in the prodigal inventions of the nineteenth century. The discipline of forbearance that it has inculcated in the human breast has so overwhelmed the disposition to profanity toward all other comparatively trifling inflictions that there is a grim satisfaction in believing that this instrument has very indirectly and quite innocently helped to develop a high grade of self-sacrificing Christian charity. All of these, however, come under the heading of really unnecessary and apparently preventable noises. Much more pronounced ones that are evidently without remedy are resignedly and philosophically borne and the very uselessness of prevention is in itself a solace of tolerance. The man who can sleep peacefully through a rattling thunder-storm loses all somnolent composure while his neighbor is filling a bath-tub, or noisily tumbling over bedroom furniture. The traveler who could composedly sleep in the berth of a limited express train would suffer the wicked suggestiveness of infant mortality while listening to the wakeful cry of a restless baby.

Strange to say, however, all these disturbers of the peace, with much show of injured innocence, are ever ready to act upon the defensive in what they consider their rights and privileges. The struggle of the brass band to maintain its boisterous hold on the street curb is still fresh in the mind of the long-suffering New Yorker. Luckily for the latter, the stuttering voice of the brazen funnel is relegated to "innocuous desuetude." Many of the street cries have been abolished after similar struggles in the cause of quiet-loving persons. Let us hope that many more reforms will follow in their wake.

It is unnecessary to allude to the widespread evil effects of noise upon the public health. If the accumulated agony of irritability could find a common voice it would dangerously jar the equilibrium of the public peace. The only mercy is that it has so many different vents through the thousand channels of individual protests that a general explosion of wrath is thereby averted. Appeals are often made to the health board for the suppression of such noises as may be construed as prejudicial to health. Practically all unnecessary noises are such, although the police catalogue places many under the designation of disorderly ones. The difficulties in the way of deciding which is which are not few. The health department, with all its arbitrary power, cannot seemingly make a politic distinction. This was shown in the case of the cruel pianist and the dying judge. Perhaps some day a proper test case may be brought for adjudication, but hardly until some enterprising neurologist can locate the noise-center and conclusively prove the direct causes of its premature exhaustion.

This subject has been further dealt with in an able manner by Dr. Augustus P. Clarke, of Cambridge, Mass., in a paper read before the section on state medicine at the Baltimore meeting of the American Medical Association, and which is published in the issue of September 14, 1895, of the association's journal. Every student of this subject should give this paper a careful reading.

The mayor of Buffalo, General Edgar B. Jewett, is a man of discipline both by education and training. It is within his province, through suggestion, advice or insistence, to modify or suppress many of the unnecessary noises that disturb, irritate or endanger many invalids. These noises also serve to drive from our prosperous city many persons of wealth who desire to live in comfort, or prevent others from locating within its boundaries. Let the mayor and the board of health conjointly take hold of this matter and see what can be done to bring relief.

